# Minority Stress and Loneliness in a Global Sample of Sexual Minority Adults: The Roles of Social Anxiety, Social Inhibition, and Community Involvement

**DOI:** 10.1007/s10508-021-02132-3

**Published:** 2022-01-27

**Authors:** Eddy M. Elmer, Theo van Tilburg, Tineke Fokkema

**Affiliations:** 1 Department of Sociology, Vrije Universiteit Amsterdam, De Boelelaan 1105, 1081 HV Amsterdam, Netherlands; 2 Netherlands Interdisciplinary Demographic Institute, KNAW/University of Groningen, The Hague, Netherlands; 3 Department of Public Administration and Sociology, Erasmus University Rotterdam, Rotterdam, Netherlands

**Keywords:** Sexual orientation, Lesbian/gay/bisexual, Stigma, Harassment, Discrimination

## Abstract

Research suggests that loneliness among sexual minority adults is associated with marginalization, but it is unclear which processes may underlie this relationship. This cross-sectional study examined five possibilities: stigma preoccupation, internalized homonegativity, sexual orientation concealment, social anxiety, and social inhibition. The study also examined the possible protective role of LGBTQ community involvement. Respondents were 7856 sexual minority adults aged 18–88 years from 85 countries who completed an online survey. Results of structural equation modeling indicated that marginalization was positively associated with both social and emotional loneliness, and that part of this relationship was indirect via proximal minority stress factors (especially stigma preoccupation) and, in turn, social anxiety and social inhibition. Moreover, while LGBTQ community involvement was associated with greater marginalization, it was also associated with lower levels of proximal stress and both forms of loneliness. Among those who were more involved in the LGBTQ community, the associations between marginalization and proximal stress were somewhat weaker, as were those between stigma preoccupation and social anxiety, and between social inhibition and social loneliness. In contrast, the associations between concealment and social anxiety were somewhat stronger. Model fit and patterns of association were similar after controlling for the possible confounding effect of dispositional negative affectivity, but several coefficients were lower. Findings underscore the continuing need to counter marginalization of sexual minorities, both outside and within the LGBTQ community, and suggest possible avenues for alleviating loneliness at the individual level, such as cognitive-behavioral interventions targeting stigma preoccupation and social anxiety.

## Introduction

Humans are a fundamentally social species with a basic need to belong and a strong drive for intimacy and companionship (Baumeister & Leary, 1995; Cacioppo & Patrick, 2008). As such, they are prone to loneliness—the painful emotional response resulting from a mismatch between actual and desired relationships (de Jong Gierveld, 1987). Loneliness can present in at least two forms: social and emotional (Hawkley et al., 2005; Weiss, 1973). *Social loneliness* is the perceived absence of a satisfactory social network—a broad circle of friends, family, colleagues, and neighbors who provide support and a sense of belonging, companionship, and community. *Emotional loneliness*, also known as intimate loneliness, is the perceived absence of a satisfying, meaningful relationship with a significant other—a close, secure attachment figure like a spouse or best friend who not only provides emotional support but also affirms our value as a person. Emotional loneliness can be accompanied by feelings of desolation or abandonment. Unlike isolation, loneliness is *subjective*: depending on one’s unique needs for social connection and intimacy, and on how the quality of relationships is perceived, a person may feel lonely on their own, in a relationship, or in a group.

Like hunger, thirst, and physical pain, loneliness evolved to alert us when our basic needs are lacking and prompts us to take corrective action (Cacioppo & Patrick, 2008; Cacioppo et al., 2013). Although beneficial in terms of motivating social connection, unresolved loneliness is a risk factor for morbidity and early mortality (Cacioppo & Cacioppo, 2014; Holt-Lunstad et al., 2015). Consequently, it is an emerging public health issue drawing attention from governments around the world (e.g., Elmer, 2018; Kodama, 2021; National Seniors Council of Canada, 2017; United Kingdom Government, 2018).

Risk factors for loneliness are diverse and include a small social network (if a larger one is desired), poor-quality relationships, attachment insecurity, unrealistic expectations, personality traits like negative affectivity and neuroticism, mental health issues, disability, and genetics (de Jong Gierveld et al., 2018; Elmer, 2018; Lim et al., 2020). Loneliness can become self-reinforcing by increasing social withdrawal; passivity; hypervigilance for social threat and conflict; negative interpretation of neutral or ambiguous social cues; unfavorable appraisals of self and others; hostility; and other negative emotions and behaviours (Cacioppo & Hawkley, 2009; Cacioppo et al., 2017; Mund & Johnson, 2021; Mund & Neyer, 2016, 2019; Qualter et al., 2015; Segel-Karpas & Ayalon, 2020; Spithoven et al., 2017; van Winkel et al., 2017). These dynamics can elicit negative perceptions and reactions by others and make *them* feel lonely, too (Lieberz et al., 2021; Simon & Walker, 2018). In this way, loneliness can spread within social networks (Cacioppo et al., 2009).

### Loneliness and Sexual Orientation

Belonging to a sexual minority is a risk factor for loneliness that has received less attention in the research literature than the popular press (e.g., Bereznai, 2006; Blum, 2018; Dodwell, 2017; Hobbes, 2017). Of the few existing studies, most have focused on midlife and older adults in specific countries. Dutch researchers have found that older lesbian, gay, and bisexual (LGB) adults in the Netherlands are lonelier than their heterosexual peers (Fokkema & Kuyper, 2009; van Lisdonk & Kuyper, 2015). Others have found that midlife and older LGB adults in North America are more likely to feel lonely and fear growing old and dying alone (AARP Foundation, 2018; Angus Reid Institute, 2019; Hsieh & Liu, 2021; MetLife, 2010; SAGE USA, 2014). Doyle and Molix (2016) found a similar sexual orientation disparity in a mixed-age sample, as did Eres et al. (2021), but their results were based on American and Australian samples, respectively. The current study extends this small literature by examining loneliness among sexual minority adults across a wider range of ages and countries.

### Loneliness and Minority Stress

The sexual orientation disparity in loneliness may be due in part to sociodemographic differences. Sexual minorities are more likely to be single, childless, living alone, in less frequent contact with families of origin, and generally at greater risk for isolation (Angus Reid Institute, 2019; Eres et al., 2021; European Union Agency for Fundamental Rights, 2014; Fokkema & Kuyper, 2009; Hsieh & Liu, 2021; Lunn et al., 2017; MetLife, 2010; SAGE USA, 2014).

An equally important reason may be *minority stress*: the negative impact of living with a stigmatized identity (Meyer, 1995, 2003). There are two types of minority stress. *Distal stressors* are prejudicial experiences like discrimination, harassment, and violence. *Proximal stressors* are internal, subjective reactions to distal stressors. One common proximal stressor is *concealment*—the desire to hide one’s sexual orientation from others. Another proximal stressor is *internalized homonegativity*, also known as *self-stigma*, which is the internalization of negative societal attitudes toward non-heterosexual orientations. Sexual minorities who internalize these attitudes—even if they are subtle and implicit—can subsequently develop feelings of guilt, inferiority, and shame. A third proximal stressor is *sexual orientation rejection sensitivity*—the tendency to anxiously or angrily expect, readily perceive, and intensely react to rejection based on one’s sexuality (Feinstein, 2020; Pachankis et al., 2008). This enduring disposition, rooted in early experiences of rejection, may begin as a form of self-protection but become maladaptive when it is activated indiscriminately and in situations that are likely benign (Feinstein, 2020). According to minority stress theory, both distal and proximal stressors help explain why sexual minorities are at increased risk for problems like anxiety and depression (Meyer, 1995, 2003).

Minority stress may be particularly relevant to loneliness. Discrimination and harassment can increase proximal stressors that interfere with the formation and maintenance of satisfying relationships. Those who desire to conceal their sexual orientation to avoid negative reactions may self-isolate and have difficulty finding other sexual minority friends or partners. They may be guarded and reluctant to discuss their personal lives, resulting in superficial, distant interactions that undermine intimacy and connection (Newheiser & Barreto, 2014). Concealment may prevent couples from engaging in shared activities in public, and the constant fear of discovery may create stress that ultimately impacts relationship quality (Knoble & Linville, 2010; Pepping et al., 2019). Sexual minorities who anxiously or angrily expect rejection, and readily perceive it even in the presence of minimal or ambiguous cues, may become avoidant, aloof, hostile, or rejecting—defensive behavior that drives people away (Feinstein, 2020; London et al., 2007; Norona & Welsh, 2016; Watson & Nesdale, 2012; Zimmer-Gembeck et al., 2016). Internalized beliefs that non-heterosexual relationships are inferior or dysfunctional could motivate relationship avoidance; reduce trust, commitment, and intimacy; and increase relationship conflict and dissatisfaction (Cao et al., 2017; Downs, 2012; Doyle & Molix, 2015; Frost & Meyer, 2009). Such beliefs could also lead to unrealistically high relationship standards—an over-compensation that narrows one’s pool of potential friends and partners, and strains existing relationships (Downs, 2012). Holding negative views of one’s sexual orientation can also make one less attractive as a potential friend or relationship partner (Frost & Meyer, 2009). Even absent proximal stress, marginalization could increase loneliness by making one feel different, misunderstood, or estranged from others. Minority stress might also be exacerbated by loneliness itself, as the latter can increase hypervigilance for social threat and conflict; negative appraisal of others; and social withdrawal (Cacioppo & Hawkley, 2009; Qualter et al., 2015; Segel-Karpas & Ayalon, 2020; Spithoven et al., 2017; van Winkel et al., 2017). Thus, minority stress and loneliness may be mutually reinforcing.

These dynamics may explain some of the empirical findings linking sexual minority stress with loneliness. Mereish and Poteat (2015) found that loneliness is associated with marginalization, internalized homonegativity, and concealment among sexual minority adults in the USA. Similar results were found in a more global sample (Mereish et al., 2017). In a study of Chinese gay men, disclosure of sexual orientation was associated with lower levels of loneliness (Jiang et al., 2019). In American studies of midlife and older sexual minorities, loneliness was associated with internalized homonegativity (Jacobs & Kane, 2012; Kim & Fredriksen-Goldsen, 2016). In two Dutch samples, older LGB adults who experienced rejection (or anticipated experiencing it) tended to be lonelier, and minority stress accounted for variance in loneliness over and above partner status, social network size, health, and self-esteem (Kuyper & Fokkema, 2010; van Lisdonk & Kuyper, 2015). Finally, a prospective study of midlife and older LGB adults in the UK found that perceived discrimination predicted loneliness six months later (Jackson et al., 2019). In the current study, we sought to extend these findings to a larger, international sample.

While past studies have focused on overt marginalization like discrimination, verbal harassment, and violence, fewer have examined less overt forms like microaggressions—subtle, seemingly innocuous, and often ambiguous words or behaviors that, intentional or not, may convey bias, disrespect, or hostility toward marginalized groups (Lilienfeld, 2017; Nadal et al., 2016). Although more subtle, these forms of marginalization may affect self-esteem and mood (Dyar et al., 2020; Wegner & Wright, 2016; Wright & Wegner, 2012). It is unknown if they are also associated with loneliness. Accordingly, our study examines both overt marginalization and microaggressions, providing a more comprehensive assessment of distal minority stress. Use of a less comprehensive measure may partly explain why one study failed to find a link between marginalization and loneliness (Doyle & Molix, 2016).

We also examined *sexual orientation stigma preoccupation*, a less commonly examined proximal stress factor. This is the maladaptive hyper-awareness of the stigmatization of one’s sexual orientation (Dyar et al., 2018). Stigma preoccupation is considered the cognitive-affective consequence of rejection sensitivity: those who are rejection-sensitive may become preoccupied and overly worried about how others perceive and judge them based on their sexual orientation (Dyar et al., 2018). Research has found that both rejection sensitivity and stigma preoccupation play a role in the association between marginalization and anxiety, including social anxiety (Dyar et al., 2018; Feinstein et al., 2012; Timmins et al., 2020). Stigma preoccupation may also be associated with loneliness, perhaps by increasing self-consciousness and self-focus—traits that can be unattractive to others (Cacioppo et al., 2017). This would be in line with prospective studies finding that rejection sensitivity predicts both social anxiety and loneliness in the general population (London et al., 2007; Zhou et al., 2020).

### Social Anxiety and Inhibition in the Relationship Between Minority Stress and Loneliness

The original formulation of minority stress theory did not consider the specific pathways by which distal and proximal stress may confer risk for psychopathology. Extending the theory, Hatzenbuehler (2009) proposed several general psychological processes that link distal stress to psychopathology. Examples include pessimism, hopelessness, negative self-schemas, and emotional dysregulation. This psychological mediation framework aimed to show how minority stress “gets under the skin.” Further extensions of this framework proposed the mediating roles of sexual orientation rejection sensitivity (Feinstein, 2020) and rumination (Timmins et al., 2020).

Few studies of minority stress and loneliness have examined the general psychological processes by which minority stress might lead to loneliness. An exception is Mereish and Poteat (2015), who found evidence for the role of shame. Our study addresses this shortcoming by examining the possible roles of social anxiety and social inhibition. *Social anxiety* is fear and worry of negative evaluation by others—either in specific situations or more globally—and can increase self-consciousness, inferiority, shame, and difficulty reading social cues (Knowles et al., 2015; Schneider et al., 2002). Notably, social anxiety is more prevalent among sexual minorities compared to others and is also associated with minority stress (Mahon et al., 2021). *Social inhibition* is the tendency to suppress expression of emotions or behaviors in order to avoid negative judgments (Asendorpf, 1993; Gest, 1997). A prospective study with the general population found that social anxiety predicts later loneliness (Lim et al., 2016). A partially prospective study, also with the general population, suggested that social inhibition predicts low sense of belonging (de Moor et al., 2018).

Cross-sectional studies have found that marginalization, concealment, internalized homonegativity, and sexual orientation rejection sensitivity are all associated with social anxiety (Cohen et al., 2016; Feinstein et al., 2012; Mereish & Poteat, 2015; Pachankis et al., 2018). One study found that marginalization (specifically parental rejection), internalized homonegativity, and rejection sensitivity are all associated with social unassertiveness, a factor closely related to social inhibition (Pachankis et al., 2008). Another study found that marginalization is indirectly associated with social anxiety via internalized homonegativity and rejection sensitivity (Feinstein et al., 2012). Finally, a prospective daily diary study with young gay and bisexual men found that parental disapproval predicts public self-consciousness, and that self-consciousness and concealment mediate the link between parental disapproval and general anxiety (Pachankis & Bernstein, 2012). Given the findings above, it is plausible that minority stress could contribute to social anxiety and inhibition, which may in turn increase loneliness by motivating self-protective social withdrawal. Accordingly, we examine the roles of social anxiety and inhibition in the relationship between minority stress and loneliness.

### Protective Role of LGBTQ Community Involvement

While several minority stress factors could increase loneliness, other factors could reduce minority stress, or at least its contribution to loneliness. One such factor is LGBTQ community involvement—having a supportive network of sexual minority friends or being a member of LGBTQ clubs, sports teams, or organizations (Meyer, 2003). Kuyper and Fokkema (2010) found that community involvement is negatively associated with loneliness among older Dutch LGB adults. The same relationship was found among Latino-American gay men living with HIV (Ramirez-Valles et al., 2005). Community involvement is also negatively associated with concealment motivation and internalized homonegativity (Bissonette & Syzmanski, 2019; Foster-Gimbel et al., 2020; Frost & Meyer, 2009; Kuyper & Fokkema, 2010; Velez & Moradi, 2016). Moreover, in studies of sexual minority adults in the USA and Hong Kong, associations between marginalization, internalized homonegativity, and poor psychological well-being were lower among people who were *higher* in various forms of LGBTQ community involvement (Chan & Mak, 2021; Salfas et al., 2019; Toomey et al., 2011; Velez & Moradi, 2016; see also Ramirez-Valles et al., 2005).

Although community involvement might *increase* marginalization by increasing one’s exposure as a sexual minority (Bissonette & Syzmanski, 2019; LeBeau & Jellison, 2009), it offers several possible benefits that could concurrently *reduce* proximal stress and buffer its impact on loneliness. LGBTQ community involvement can provide a supportive social network which increases comfort with one’s sexuality, validates one’s experiences of marginalization, promotes adaptive reappraisals of marginalization, and fosters a sense of acceptance, belonging, and understanding (Cox et al., 2010; Kuyper & Fokkema, 2010; LeBeau & Jellison, 2009; Roberts & Christens, 2021; Velez & Moradi, 2016; Westefeld et al., 2001). Community involvement may also promote empowerment, personal control, self-efficacy, and self-esteem (Heath & Mulligan, 2008; LeBeau & Jellison, 2009; Ramirez-Valles et al., 2005; van Lisdonk & Kuyper, 2015; Wernick et al., 2014; Westefeld et al., 2001). These factors are prospectively associated with decreased loneliness (Newall et al., 2014; Spithoven et al., 2017; Vanhalst et al., 2013). Given these lines of evidence, we examine whether LGBTQ community involvement is negatively associated with loneliness in a global sample. We also examine whether it is positively associated with marginalization, but negatively associated with proximal stress, and whether the links between marginalization, proximal stress, social anxiety/inhibition, and loneliness are weaker among those who are more community-involved.

### Dispositional Negative Affectivity as a Possible Confounding Factor

Several researchers argue that studies of subjectively reported discrimination often overlook the confounding effect of personality traits (Bailey, 2020; Lilienfeld, 2017). Perceptions of marginalization and its subjective impact, along with rejection sensitivity and loneliness, may all be partly influenced by *general negative affectivity* (NA). This is an inherent and enduring disposition to experience dysphoria, anxiety, and irritability across situations (Denollet, 2005; Watson & Clark, 1984). People high in NA have a negative view of themselves and others, and are more likely to perceive benign or ambiguous situations in a negative or threatening manner (Brief et al., 1988; Watson & Clark, 1984). In addition, a longitudinal study with adults found that negative affectivity predicts rejection sensitivity in early childhood (Araiza et al., 2020). In addition, a longitudinal study with adults found that neuroticism—closely related to NA—predicts loneliness over 15 years (Mund & Neyer, 2016). We examine whether dispositional negative affectivity may confound the relationship between minority stress and loneliness, especially since our measures of marginalization are affectively laden (e.g., measuring not only the frequency of microaggressions but also how much a person is bothered by them).

### The Current Study

**Fig. 1:**
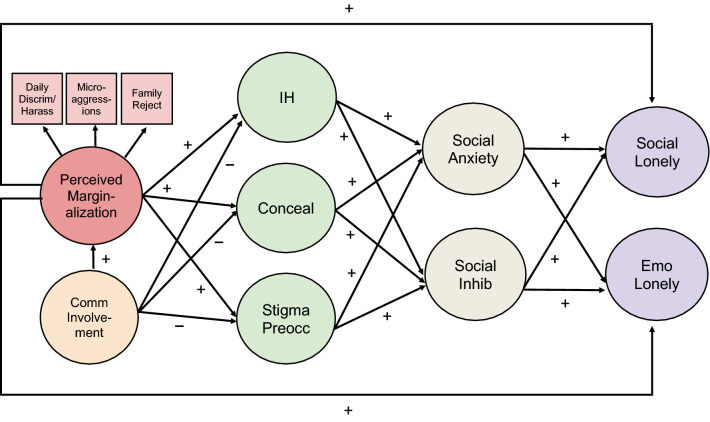
Proposed structural equation model linking marginalization and loneliness*. Notes:* Daily Discrim/Harass = everyday discrimination and harassment, Family Reject = family rejection, Comm Involvement = community involvement, IH = internalized homonegativity, Conceal = concealment, Stigma Preocc = stigma preoccupation, Social Inhib = social inhibition, Social Lonely = social loneliness, Emo Lonely = emotional loneliness. For ease of presentation, direct associations between community involvement/proximal stress and both forms of loneliness are not shown, nor are direct associations between marginalization/community involvement and social anxiety/inhibition. We also do not show the a priori correlations between social anxiety and inhibition, and between both forms of loneliness

We proposed a structural equation model (SEM) which includes direct and indirect associations between distal minority stress (i.e., marginalization), proximal minority stress (i.e., internalized homonegativity, concealment, stigma preoccupation), social anxiety, social inhibition, community involvement, and loneliness (Fig. 1). Unlike previous studies, we examine both social and emotional loneliness, as they may have different associations with minority stress, social anxiety, inhibition, and community involvement. We propose several hypotheses based on our conceptual model:

#### H1:

Marginalization (defined as perceived experiences of everyday discrimination/harassment, microaggressions, and family rejection) is positively associated with both social and emotional loneliness.

#### H2:

Marginalization is positively associated with internalized homonegativity, concealment of sexual orientation, and stigma preoccupation.

#### H3:

Internalized homonegativity, concealment, and stigma preoccupation are positively associated with social anxiety and inhibition, and with social and emotional loneliness (for simplicity, Fig. 1 only shows associations with social anxiety/inhibition).

#### H4:

Social anxiety and inhibition are positively associated with social and emotional loneliness.

#### H5:

Marginalization is positively associated with social and emotional loneliness via indirect relationships with internalized homonegativity, concealment, and stigma preoccupation.

#### H6:

Marginalization is positively associated with social and emotional loneliness via indirect relationships with internalized homonegativity, concealment, and stigma preoccupation, and, in turn, via social anxiety and social inhibition (i.e., serial indirect associations).

#### H7:

LGBTQ community involvement is positively associated with marginalization.

#### H8:

LGBTQ community involvement is negatively associated with internalized homonegativity, concealment, stigma preoccupation, and both forms of loneliness (for simplicity, Fig. 1 does not show direct associations with loneliness).

#### H9:

All associations in the model are weaker among those who are higher in LGBTQ community involvement (i.e., moderation).

#### H10:

All associations decline after controlling for dispositional general negative affectivity.

## Method

### Participants

Our sample was 7856 sexual minority adults from 85 countries (30% from non-Western countries), with 20% living in suburban areas and 22% in small towns or rural areas. They were aged 18 − 88 (*M* = 32.91, *SD* = 14.31, *Mdn* = 27), with 52% identifying as men, 38% as women, 4% as transgender men or women, and 6% as other (with non-binary as the most common fill-in response). Fifty-six percent identified as gay/lesbian/homosexual, 15% as mostly gay/lesbian/homosexual, 17.5% as bisexual, 7% as pan/polysexual, and 4.5% as queer. Thirty percent identified as non-White and a little over half (53%) were partnered. Table 1 provides detailed demographic characteristics.

**Table 1:** Demographic characteristics

Characteristic	*n*	Percent or Average
Age	7856	*M* = 32.91 *SD* = 14.31 *Mdn* = 27 *Range* = 18–88
Sex assigned at birth		
Male	4282	54.5%
Female	3572	45.5%
Gender identity		
Male	4073	52%
Female	3017	38%
Transgender: male-to-female*	120	1.5%
Transgender: female-to-male*	206	2.5%
Other, including non-binary	440	6%
Sexual orientation		
Gay/lesbian/homosexual	4417	56%
Mostly gay/lesbian/homosexual	1177	15%
Bisexual	1362	17.5%
Pan/polysexual	543	7%
Queer	357	4.5%
Ethnoracial identity		
White	5481	70%
Latinx/Hispanic	949	12%
Asian/Pacific Islander	597	7.5%
African/Black/Caribbean	192	2.5%
Arab/Middle Eastern	60	1%
Indigenous/Aboriginal	62	1%
Mixed/Multi	455	6%
Other	60	1%
Global region		
North America	2979	38%
Latin America and Mexico	1080	14%
UK and Ireland	871	11%
Western Europe (Other)	815	10.5%
Eastern Europe	673	8.5%
Asia	558	7%
Australia and New Zealand	466	6%
South Africa	348	4.5%
Middle East and North Africa	66	1%
Geographic type		
Urban	4570	58%
Suburban	1569	20%
Rural/Small Town	1694	22%
Partnered	4136	53%
Comfort with Income (1 = *Not at all comfortable*; 7 = *Extremely comfortable*)	7550	*M* = 4.19 *SD* = 1.82 *Mdn* = 4

^*^Includes individuals whose gender identity is discordant with their sex assigned at birth, but who do not identify as transgender. *N*s may not equal 7,856 due to small proportions of missing responses. Percentages may not equal 100% due to rounding

### Data Collection

After receiving ethics approval, we conducted an online survey during summer and fall 2016. To reach a global audience, we used social media click-ads, asking users aged 18+ to participate in a study about “LGBTQ social relationships and well-being” (a minority of ads also used the term “mental health”). Some 78% of respondents in the current analysis found the survey via demographically targeted Facebook ads, 4% via targeted Instagram ads, 5% via notices on LGBTQ Facebook groups, and the rest via other websites (e.g., Tumblr, Reddit) or email. The paid ads were targeted to users who had explicitly indicated in their profile, either publicly or privately, that they were interested in people of the same gender or in LGBTQ topics. To reach people not as open about their sexual orientation, we targeted the same ads for a limited time to people who specified no sexual preference at all. We also ran a generic ad referencing “social relationships and well-being,” with no mention of sexual orientation; 4.5% of respondents in the current analysis were reached this way. To maximize diversity, advertising was done purposively to ensure sufficient respondents of various ages, ethnicities, relationship statuses, political orientations, and regions. Ads were run prior to Facebook’s 2018 guidelines that removed the ability to target ads by sexual orientation and race.

Social media advertising has been used successfully in previous studies of hard-to-reach populations, including sexual minorities (King et al., 2014; Thornton et al., 2016). Although not without its risks and limitations (Littler & Joy, 2021), this method allows recruitment of LGBTQ sub-populations that are underrepresented in research, including older adults, those in non-Western and rural regions, and those not highly involved in the LGBTQ community. The latter is important because people higher in community involvement—who are often the primary source for minority stress research—may differ in meaningful ways from those who are less community-involved (e.g., in real or perceived exposure to minority stress, or motivation for participating in research; Kuyper et al., 2016; Meyer & Wilson, 2009).

Participants completed the survey anonymously using Qualtrics. Given its length, participants were able to pause and resume within two weeks. Ninety-four percent completed on the same day. To flag inattentive responding, we placed four “attention check” items at equal intervals throughout the survey (e.g., “Select answer 3 for this question”). Of those who completed most of the survey, the failure rate for the first three attention checks was 2 − 2.5%. For the final attention check, failure was higher, at 13.5%. We attribute this to its location at the end of the survey (i.e., fatigue) and its suboptimal placement in a question matrix where it was easy to miss.

To reduce comprehension problems, we advertised to individuals whose social media profiles indicated that they understood English. We also examined the internal consistency of responses across countries and races/ethnicities; results suggested that individuals comprehended questions in a similar manner.

As an incentive, participants could enter a draw for one of several Amazon gift cards ranging from $20 − $200 USD or a donation to a charity of their choice.

### Screening and Eligibility

A total of 23,458 respondents completed the screening section of the survey (informed consent and basic demographics). Of these, 15,830 were self-identified sexual minorities, of whom 14,449 chose to start the main part of the survey (i.e., detailed questions about sexuality). This does not include 986 people who identified as questioning, asexual, or other. We did not include these individuals in the current analysis because they were not shown all of the required minority stress scales. As most of these scales refer to sexual orientation (e.g., being LGBTQ, being attracted to people of the same gender), we felt that many of these respondents would find the scales to be inapplicable, confusing, or inappropriate. Moreover, for those who had selected “other” and typed a response, it was not possible to determine if they were sexual minorities and thus to present them with all of the minority stress scales. We also excluded those who identified as straight/heterosexual or mostly straight/heterosexual (*n* = 6642); they had completed many of the same scales as sexual minorities (e.g., loneliness, social anxiety), but not all of the ones required for the current analysis. Based on their Kinsey scores, some of these respondents could be classified as LGBTQ, but we did not include them because they did not specifically identify as sexual minorities.

### Survey Completion

Of the initial 14,449 sexual minorities who started the main part of our survey, 10,377 (72%) completed one-third of the survey (including the concealment, community involvement, and loneliness scales); 8563 (60%) completed half the survey (including the social anxiety, inhibition, and negative affectivity scales); and 7974 (55%) completed ≥ 80% of the survey (including the internalized homonegativity and stigma preoccupation scales, and at least the first marginalization scale—microaggressions). This completion rate is similar to that of other large online studies of loneliness (AARP, 2018), sexuality (BBC Internet Study; Reimers, 2007), and minority stress (Brewster et al., 2013; Community-Based Research Centre, 2017; Meyer et al., 2020). Those who quit prematurely (i.e., completed < 80% of the survey) appeared to do so because of survey length (median completion time = 73 min), technical problems on smartphones, pausing and forgetting to resume the survey later, or trying to resume on a different device. Completers were slightly older (*M* age = 32.93, *SD* = 14.31) compared to non-completers (*M* age = 29.72, *SD* = 12.14), *t*(14,410) = 14.56, *p* < .001. Queer and pan/polysexual respondents were more likely to complete compared to others (62% versus 52% − 56%), *χ*^2^(4) = 36.49, *p* < .001. There were also notable differences by ethnoracial identity: 61% of White respondents completed compared to 49% of Latinx/Hispanic individuals, 36% of Asians, and 51% of other races/ethnicities, *χ*^2^(3) = 407.28, *p* < .001. Those who had started on a mobile device were also less likely to complete compared to those who had used a desktop computer (46% versus 64%), *χ*^2^(1) = 452.40, *p* < .001 There were no meaningful differences in loneliness between completers and non-completers.

Of the 7974 respondents who completed at least 80% of the survey (our minimum acceptable completion rate), we excluded from the current analysis those who were under age 18 (*n* = 9), who specified no age (*n* = 15), who failed more than two attention checks (*n* = 62), who appeared to provide duplicate or nuisance responses (*n* = 18), or who were missing more than 20% of item-level data across all scales (*n* = 14). We did not exclude the small number of respondents with user-missing values on the marginalization, concealment, or community involvement scales, as many of these values appear to have been “not applicable” answers that respondents failed to record as such. Other respondents were missing values because they had quit the survey before completing the final two marginalization scales; we handled their missing data using imputation (see Missing Data section). The final sample was 7856 individuals.

### Measures

#### Loneliness

We used the 11-item de Jong Gierveld Loneliness Scale (de Jong Gierveld & Kamphuis, 1985; de Jong Gierveld & van Tilburg, 2021). Five positively worded items measure feelings of social embeddedness and sense of belonging (e.g., “There are plenty of people I can lean on when I have problems”). Six negatively worded items measure feelings of desolation and longing for an intimate attachment (e.g., “I miss having a really close friend”). Given the shame surrounding loneliness, as well as individuals’ varying interpretations of the term, the words “lonely” and “loneliness” do not appear in the scale because they might lead to under-reporting, especially among men (Badal et al., 2021; Nicolaisen & Thorsen, 2014; Victor et al., 2005).

Respondents indicated their agreement with each statement on a 5-point Likert-type scale (1 = *Yes!*, 2 = *Yes*, 3 = *More or less*, 4 = *No*, 5 = *No!*). Disagreeing with a positive item (*No!*, *No*, and *More or less*) indicated social loneliness and was assigned a score of 1. Agreeing with a negative item (*Yes!*, *Yes*, and *More or less*) indicated emotional loneliness and was assigned a score of 1. Scores were summed to yield separate totals for social and emotional loneliness. Although the scale was designed to be scored dichotomously (each item receiving 0 or 1), it can also be scored continuously. Our results were similar regardless of the method, but we report values based on the original method in order to facilitate comparison with previous studies.

The Loneliness Scale is valid and reliable for use in different countries and among different ethnicities (de Jong Gierveld & Van Tilburg, 2010; Uysal-Bozkir et al., 2017; van Tilburg & Fokkema, 2021; van Tilburg et al., 2004). In the current study, internal consistency reliability, as measured by McDonald’s omega (ω), was good: .87 for emotional loneliness and .86 for social loneliness (omega, also called *omega total*, is interpreted similarly to alpha; McNeish, 2018).

#### Marginalization

We used three measures of marginalization. Microaggressions were measured with the Second-Class Citizen subscale of the Homonegative Microaggressions Scale (HMS; Wegner & Wright, 2016; Wright & Wegner, 2012). This subscale refers to experiences that can make sexual minorities feel like lesser members of society. Respondents indicated how often in the last 12 months they have experienced each of eight events (e.g., “People telling you to act differently at work, school, or other professional settings in order to hide your sexual orientation”). Answers were recorded on a five-point Likert-type scale (1 = *Never/Not applicable*; 5 = *Constantly*). Unlike other scales, the HMS also asks respondents how much each experience bothered them (1 = *Not at all/not applicable*; 5 = *A great deal*). For each item, the two responses are multiplied; a total scale score is calculated as the mean of the products.

Although the HMS includes four subscales, we chose the Second-Class Citizen subscale to minimize survey length and because it contains items that are in the middle of the continuum of microaggressions. By contrast, the first subscale (Assumed Deviance) reflects intentional, hostile experiences (e.g., “How often have people assumed you were a pervert or deviant?”), while the other two (Assumptions of Gay Culture; Stereotypical Knowledge and Behavior) reflect more subtle experiences without intention to harm (e.g., “How often have people assumed you were knowledgeable in stereotypically gay tasks, like interior design for men or carpentry for women?”). The full HMS exhibits good factor structure, construct validity, and reliability, and the Second-Class Citizen Subscale is strongly correlated with internalized homonegativity, acceptance concerns, and other proximal stress factors (Wegner & Wright, 2016; Wright & Wegner, 2012). In the current study, reliability for this subscale was good (ω = 0.83).

We measured everyday discrimination/harassment and family rejection using the Daily Heterosexist Experiences Questionnaire (DHEQ; Balsam et al., 2013). Respondents indicate how often in the last 12 months they have experienced various heterosexist events, which are divided into nine subscales. For the current analysis, we used the 6-item Discrimination/Harassment subscale (e.g., “Being verbally harassed by strangers because you are LGBT”; “Being treated unfairly in stores or restaurants because of your sexual orientation”). We also used the 6-item Family of Origin subscale (e.g., “Your family avoiding talking about your LGBT identity”). We felt these subscales captured the more common forms of overt marginalization. We also administered the Victimization subscale, focusing on violent events, but did not use it in the current analysis due to very low item endorsement. The other subscales are only applicable to specific subgroups (e.g., parents, people with HIV) or reflect constructs that we measured using other scales (e.g., Isolation, Vigilance).

To focus on discrimination and harassment related to sexual orientation and not gender identity, we replaced “LGBT” with “sexual orientation” (e.g., “Being verbally harassed by strangers because of your sexual orientation”). For consistency, we also modified the response options to match those of the HMS, with subscale totals calculated in the same manner.

The DHEQ exhibits excellent content, construct, and criterion validity, and good reliability (Balsam et al., 2013; Morrison et al., 2016). In the current sample, reliability was good for Discrimination/Harassment (ω = 0.83) and Family of Origin (ω = .79).

#### Internalized Homonegativity

We used six modified items from the 11-item Personal Homonegativity subscale of Mayfield’s (2001) Internalized Homonegativity Inventory (IHNI). This subscale assesses negative emotions and attitudes about one’s sexual orientation (e.g., “I feel ashamed of my homosexuality”). To be more inclusive, we changed “my homosexuality” to “being attracted to people of the same gender” (here we did not use “sexual orientation” because, for purposes of another study, the scale was also administered to individuals with at least some same-gender desire but who consider themselves heterosexual; i.e., individuals who are unlikely to indicate feeling ashamed of being heterosexual). Responses were recorded on a six-point Likert-type scale (1 = *Strongly disagree*; 6 = *Strongly agree*). A mean was calculated, with higher scores indicating greater internalized homonegativity.

Although the IHNI has two other subscales—Gay Affirmation and Morality of Homosexuality—we chose Personal Homonegativity to minimize survey length and because the items about negative emotions (e.g., shame, embarrassment, resentment) are most relevant to social anxiety, inhibition, and loneliness. In contrast, the Gay Affirmation subscale is about LGBTQ pride, and the Morality of Homosexuality subscale is about general attitudes rather than emotions.

The full scale demonstrates good validity and reliability (Choi et al., 2017). Although initially developed for gay men, the INHI is applicable to other groups and has been used in mixed-gender studies with modified wording (e.g., Kuyper & Bos, 2016). Due to space constraints, we chose the six items from the Personal Homonegativity subscale that best represented the construct and that exhibited the highest factor loadings in previous studies. Reliability of the reduced subscale was excellent (ω = .91).

#### Sexual Orientation Concealment

We used a modified version of the Concealment subscale of the Nebraska Outness Scale (NOS; Meidlinger & Hope, 2014). Respondents specified how often they avoid indicating or implying their sexual orientation when interacting with five groups: immediate family, extended family, friends, coworkers/associates, and strangers (0 = *Never*; 10 = *Always*). Examples of concealment include changing one’s mannerisms or avoiding topics related to one’s sexual orientation (e.g., discussion about same-gender partners). Unlike other scales, the NOS lacks a “not applicable” option (e.g., for people with no extended family). We added this option, which was selected by up to 5.5% of respondents, and assigned it a score of zero, given that the absence of a particular group of people in one’s life reduces the frequency and burden of concealment. A mean is calculated, with higher scores indicating greater concealment. As with the IHNI, we replaced “sexual orientation” with “attraction to people of the same gender.”

The NOS has a second subscale that also measures *disclosure*: how much one thinks other people are aware of one’s sexual orientation. We chose the Concealment subscale because it is more strongly associated with mental distress (Meidlinger & Hope, 2014; Schrimshaw et al., 2013). The full NOS exhibits good factor structure, construct validity, criterion validity, and reliability (Meidlinger & Hope, 2014). In the current study, reliability for the Concealment subscale was good (ω = .82).

#### Stigma Preoccupation

We used a modified version of the three-item Acceptance Concerns subscale of the Lesbian, Gay, and Bisexual Identity Scale (LGBIS; Mohr & Kendra, 2011). The items assess a person’s concerns about how they are perceived and judged because of their sexual orientation (e.g., “I often wonder whether others judge me for my sexual orientation”). Similar to the modified IHNI and NOS, we changed “my sexual orientation” to “my attraction to people of the same gender.” Responses were recorded on a six-point Likert-type scale (1 = *Strongly disagree*; 6 = *Strongly agree*). A mean was calculated, with higher scores indicating greater stigma preoccupation. As per Dyar et al. (2018), we refer to acceptance concerns as stigma preoccupation because the latter more accurately describes the items in this subscale. The full LGBIS is valid and reliable (Mohr & Kendra, 2011) and has been tested for use in other countries (e.g., de Oliveira et al., 2012; Kemer et al., 2017). In the current study, reliability for Acceptance Concerns was good (ω = .85).

#### LGBTQ Community Involvement

We used five of seven items from the Involvement with Gay Community Scale (IGCS; Tiggemann et al., 2007). An example item is: “I am actively involved in the LGBTQ community.” Responses were recorded on a seven-point Likert-type scale (1 = *Not at all true of me*; 7 = *Extremely true of me*). A mean score was calculated, with higher scores indicating greater community involvement. The original scale has adequate validity and reliability (Tiggemann et al., 2007). Based on confirmatory factor analysis with the current sample, we deleted two reverse-keyed items with poor loadings that reduced model fit (“My closest friends are straight” and “When I go out, I generally spend time in venues *not* specifically aimed at LGBTQ individuals”). Reliability for the reduced five-item scale was good (ω = .82).

#### Social Anxiety

We used the 12-item Brief Fear of Negative Evaluation Scale—Revised, a common measure of social anxiety (BFNE–II; Carleton et al., 2006). Unlike the LGBIS, this scale measures global fear of negative evaluation (e.g., “I am afraid that people will find fault with me”). Responses were recorded on a five-point Likert-type scale (0 = *Not at all characteristic of me*; 4 = *Extremely characteristic of me*). The scale has excellent construct validity and reliability (Carleton et al., 2006, 2007). In the current study, reliability was excellent (ω = .93).

#### Social Inhibition

This was measured using the Social Inhibition subscale of the DS14 Type D Personality Scale (Denollet, 2005). Respondents indicated agreement with seven statements (e.g., “I find it hard to start a conversation”). Responses were recorded on a five-point Likert-type scale (0 = *False*; 4 = *True*). The DS14 has excellent construct validity and the Social Inhibition subscale correlates strongly with extraversion (Denollet, 2005). The scale is valid and reliable for use in many countries (e.g., Bergvik et al., 2010; Grande et al., 2010; Lim et al., 2011). In the current study, reliability was good (ω = .86).

#### Negative Affectivity

This trait was assessed using the Negative Affectivity subscale of the DS14. Respondents indicated agreement with seven items about the tendency to be dysphoric, irritable, or anxious (e.g., “I am often in a bad mood”; “I take a gloomy view of things”). Responses were recorded on a five-point Likert-type scale (0 = *False*; 4 = *True*). The subscale is reliable and correlates strongly with neuroticism (Denollet, 2005). In the current study, reliability was good (ω = .87).

#### Demographic Variables

Respondents were asked “What sex were you assigned at birth, on your birth certificate?” Options were male and female. Gender identity was assessed by asking “How do you currently describe yourself?” Options were male, female, transgender male-to-female (MTF), transgender female-to-male (FTM), and other (please specify). We dummy-coded these as female and transgender/non-binary/other, with male as the reference. Within transgender/non-binary/other, we included 99 people whose gender identity was discordant with their sex assigned at birth, but who did not specifically identify as transgender; they did not differ significantly from self-identified transgender people on most scales. Sexual orientation was assessed by asking “What do you consider your sexual orientation to be, regardless of how you describe yourself to others?” Options were straight/heterosexual; mostly straight/heterosexual; gay/lesbian/homosexual; mostly gay/lesbian/homosexual; bisexual; queer; pansexual or polysexual; questioning/not yet sure; asexual; and other (please specify). Responses were dummy-coded as mostly gay/lesbian/homosexual; bisexual; queer; and pan/ polysexual, with gay/lesbian/homosexual as the reference.

Because race/ethnicity may affect minority stress processes (e.g., Shangani et al., 2020), we asked respondents “Which of the following best describes you?” Options were Aboriginal/Native/First Nations; African/African-American/Black; Arab/Middle Eastern; Asian; Caribbean; East Indian; Hawaiian Native/Pacific Islander; Latino/Latina/Hispanic; White/Non-Hispanic White; Mixed/Multi; and Other. Responses were dummy-coded into Latinx/Hispanic, Asian, and Mixed/Multi/Other. White, the largest portion of the sample, was the reference. Following recommendations by Allen et al. (2011), we included Latino/Latina/Hispanic in the racial categories because some Latinx/Hispanic individuals, particularly in the USA, consider their ethnicity to be a race and express difficulty with separate race and ethnicity questions (Croll & Gerteis, 2019). Those who selected Other and wrote that they were White, Caucasian, European, or an ethnicity commonly considered White or “Other White” in a given country (e.g., Irish/Celtic, Ukrainian, Mediterranean, Australian, New Zealand European/Pakeha) were re-categorized into the White reference category (2%). We did this to avoid mistakenly assigning respondents to the Mixed/Multi/Other category (essentially a minority category) if it was unlikely, or unclear, that they were actually minorities.

Finally, respondents indicated their country of residence, which was dummy-coded into seven regions, with North America as the reference (UK, Western Europe, Eastern Europe, Latin America, South Africa, Australia/New Zealand, and Asia). They also indicated the type of region they lived in, which was dummy-coded into suburban and small/rural/remote, with urban as the reference. Four percent did not provide these geographic details, mostly because they had quit the survey early; their details were inferred from IP addresses, with the caveat that some may have completed the survey in a country where they do not reside.

### Analytic Procedures

#### Missing Data

For each scale, we computed mean scores if respondents answered at least 80% of items on the scale—our minimum acceptable completion rate. The same applied to computing parcel scores (see Model Specification below). Exceptions were the scales for social and emotional loneliness as well as stigma preoccupation, for which we required 100% completion, given that the first two scales are based on count data, not means, and the third contains only three items. These exceptions aside, if respondents were missing items, we computed mean scores or parcels using all available items (i.e., pro-rating). This method yields similar results to item-level multiple imputation, as long as missing data are low, sample size is large, reliability is high, and scales have at least five items (Parent, 2013).

Remarkably, 95% of respondents had no missing data and, for all but two scales, nearly 100% answered all items on each scale. Exceptions were scales for everyday discrimination/harassment and family rejection (both the frequency and distress components): for each of these scales, 3.2–3.9% of respondents were missing at least one item, with 2.5–3% missing all items on the scale. About 75% of these respondents (*n* = 161) were missing all data on these scales because they had they quit the survey early. Data were not missing completely at random (Little’s MCAR test: *χ*^2^(111) = 143.06, *p* = .022). As missing data were partially predicted by observed variables, we handled them using stochastic regression imputation in AMOS, which is sufficient for low-level missingness (Schafer, 1999). Based on response patterns and comments, most of the remaining missing items appeared to be inapplicable to respondents, yet not marked as such (e.g., questions about one’s partner or parents). We recoded these as “1” (“not at all/not applicable”).

#### Data Screening

Scale distributions were roughly normal except for marginalization, internalized homonegativity, and concealment, which exhibited skewness (+ 1.29 to + 2.91) and/or kurtosis (– 0.85 to + 11.76). This is unsurprising as most participants had low scores on these measures, consistent with other studies (e.g., Everett et al., 2019; Mereish & Poteat, 2015; van Lisdonk & Kuyper, 2015; Velez & Moradi, 2016). In fact, roughly one-third of respondents scored 1 (the lowest possible score) for everyday discrimination/harassment, family rejection, and internalized homonegativity; 20% scored 0–1 for concealment. Univariate and multivariate deviations from normality were confirmed by Q-Q plots and Mardia’s coefficient (33.78, *p* < .001).

As structural equation modeling can yield inaccurate estimates and standard errors in the presence of non-normal distributions, we log-transformed the total scores for all three marginalization scales and internalized homonegativity. This redistributed the tails somewhat, but despite lower skewness and kurtosis, the scales remained markedly L-shaped. The distribution for concealment worsened after transformation, so we used it in its original form. We retained all other transformed values and used bootstrapping to further compensate for deviations from normality. As a supplementary step, we conducted all analyses with and without log-transformations; the overall patterns of association and model fit statistics were similar between approaches, but associations were stronger using transformed values, so we report all results based on the latter.

#### Model Specification

To take full advantage of SEM’s ability to account for measurement error and thus produce more accurate parameter estimates, we created latent variables (factors), each having multiple indicators. The marginalization factor was formed using mean scores for everyday discrimination/harassment, microaggressions, and family rejection. The stigma preoccupation factor was formed using the three items from the Acceptance Concerns subscale of the LGBIS. Remaining factors were formed via parceling, which involves grouping scale items into smaller subsets. Compared to individual items, parcels can improve reliability, reduce distributional violations, and decrease the number of parameters requiring estimation (Coffman & MacCallum, 2005; Little et al., 2002).

We grouped items using the item-to-construct approach, also known as the factorial algorithm (Little et al., 2002). For most parcels, we further redistributed items to combine those with opposite skew, thus improving parcel distributions (Thompson & Melancon, 1996). However, even after this approach, parcels for internalized homonegativity were still skewed, so we log-transformed them. For all scales, we achieved similar average factor loadings within parcels. Correlations between parcels and total scale scores were all high (≥ .85), as were correlations between parcels, ranging from .60 to .85, with most over .70. While most scales were divided into three parcels, the community involvement, concealment, and social loneliness scales were divided into two, given their smaller number of items. Although three indicators/parcels per factor are ideal, two are acceptable if item communalities are high and the factors can be adequately identified (Kenny, 2012).

We linked the factors as per Fig. 1. For ease of presentation, the figure does not show the direct associations we added between community involvement/proximal stress and both forms of loneliness. While not specifically mentioned in our hypotheses, we also added direct associations between marginalization/community involvement and social anxiety/inhibition (also not shown in Fig. 1). Although adding these associations reduces model parsimony, excluding them essentially fixes them to zero, possibly inflating our theorized indirect associations (Hayes & Preacher, 2013). We also included a priori correlations between social anxiety and inhibition, and between social and emotional loneliness (not shown). We did this to depict the conceptual and empirical similarity between these variables; however, as SEM does not permit latent endogenous variables to covary, we specified these associations by correlating their residual error terms (Kenny, 2011).

#### Main Analyses

To test our model, we conducted structural equation modeling with AMOS 25 (Arbuckle, 2017). Our sample (*N* = 7856) had sufficient statistical power (≥ .80) to detect even small effects (Cohen, 1988; Westland, 2010). It was also sufficient assuming the minimum recommendation of about ten respondents per estimated parameter (Jackson, 2003).

First, we tested the validity of our measurement model to ensure that all indicator variables loaded adequately onto their respective factors. Next, we added structural paths between factors and tested all hypothesized direct and indirect associations.

To handle non-normal data, we used bias-corrected bootstrapping with 95% confidence intervals, using 5000 samples from the original dataset. We also compared results before and after exclusion of multivariate outliers (Aguinis et al., 2013; Warren et al., 2011). For all analyses, we used maximum likelihood estimation.

We used several indices to assess the fit of the measurement and structural models: the comparative fit index (CFI), Tucker-Lewis Index (TLI), root-mean-square error of approximation (RMSEA), and standardized root-mean-square residual (SRMR). Good model fit is suggested by CFI and TLI values ≥ .95 (Hu & Bentler, 1999), although some researchers suggest higher values, with ≥ .97 suggestive of good fit and ≥  .95 suggestive of adequate fit (Schermelleh-Engel et al., 2003). For RMSEA and SRMR, values ≤  .05 are suggestive of good fit (Browne & Cudeck, 1993; Hu & Bentler, 1999; Schermelleh-Engel et al., 2003).

As a measurement model may contribute strongly to overall fit even if the structural model exhibits poor fit (McDonald & Ho, 2002), we evaluated the fit of the measurement, structural, and composite models separately (O’Boyle & Williams, 2011). We also inspected standardized residual covariances for localized misfit, which can be masked by global fit indices (Tomarken & Waller, 2003). As these residual covariances tend to be inflated in large samples (Brown, 2015), we flagged values greater than 2.58 (i.e., significant at *p* < .01; Byrne, 2010). To verify if large residual covariances were due to sample size, we re-ran analyses with smaller samples comprising 25% and 50% of participants randomly drawn from the full sample.

If global fit was unsatisfactory, or standardized residual covariances indicated localized misfit, we examined modification indices for any theoretically defensible paths or error correlations that could be added (e.g., correlations between error terms of indicator variables or factors). Any such additions were added and tested iteratively. We did not delete small or statistically non-significant paths to improve fit as such paths are theoretically relevant and may be significant in replication samples or moderated mediation with different subgroups (Kline, 2016).

We checked that all associations were statistically significant and in the hypothesized directions. We also examined the size of parameters, but were cognizant that indirect associations are often small due to their multiplicative nature; these associations are even smaller when multiple serial mediators are used (Walters, 2019). Although we were primarily interested in whether indirect associations were consistent with theory, we also calculated effect sizes and expressed them in terms of relative magnitude. We did this by dividing each indirect association by the total association between marginalization and social/emotional loneliness (Preacher & Kelley, 2011; Wen & Fan, 2015).

To determine if associations in the model differed by community involvement (i.e., moderation), we added continuous latent variable interactions (e.g., marginalization x community involvement). We used double-mean-centering, which is recommended when variables are not normally distributed (Lin et al., 2010). For each latent moderation factor, we added correlated errors between product terms that shared common items. We probed statistically significant interactions using simple slopes tests, and reported associations between factors at three levels of community involvement: high (one standard deviation above the mean), medium (at the mean), and low (one standard deviation below the mean). As interaction effects in social science tend to be small, power to detect such effects can be low, even with large samples; thus, we probed interactions that were significant at the .10 level (Fairchild & MacKinnon, 2009).

Finally, to determine if trait-level general negative affectivity was a potential confound in the relationships between minority stress, social anxiety, inhibition, and loneliness, we re-ran our SEM model after adding negative affectivity as a control. It was introduced as a latent factor with three indicator parcels, each loading > .70. To see if our use of affectively laden marginalization scales may have exacerbated any confounding influence of negative affectivity, we re-ran analyses using only the *frequency* component of each scale and excluding the evaluation of subjective impact (Lilienfeld, 2017).

Models were controlled for age, gender identity, sexual orientation, ethnoracial identity, and region. While results may differ as a function of these variables, these differences will be explored in detail in a separate study, using the same data. We did not control for partner status or income as these factors may mediate the link between marginalization and loneliness; controlling for them would reduce total associations. However, to reduce model complexity, we did not explicitly model these variables as mediators. We also did not control for method of recruitment (e.g., Facebook, Instagram, other website, or email) or device type (mobile versus desktop) as they were not associated with any scale scores.

## Results

### Preliminary Analysis

As seen in Table 2, participants exhibited relatively low levels of marginalization, concealment, and internalized homonegativity, and moderate levels of stigma preoccupation, social anxiety, inhibition, negative affectivity, and community involvement. Using cut-off values based on combined social and emotional loneliness scores (de Jong Gierveld & van Tilburg, 2021), 47% of respondents were moderately lonely, 18% very lonely, and 10% severely lonely. All zero-order correlations were significant and in the expected directions.

**Table 2:** Descriptive statistics and Pearson’s correlations between major study variables

	1	2	3	4	5	6	7	8	9	10	11	12
1. Discrim/harass	–											
2. Microaggress	.57	–										
3. Family reject	.46	.48	–									
4. Comm involve	.11	.11	.04	–								
5. IH	.21	.21	.21	− .24	–							
6. Concealment	.10	.19	.20	− .24	.44	–						
7. Stigma	.34	.37	.32	− .17	.55	.44	–					
8. Social anxiety	.24	.25	.18	− .06	.33	.25	.43	–				
9. Social inhib	.10	.13	.09	− .17	.22	.22	.25	.41	–			
10. Emo lonely	.18	.20	.17	− .13	.29	.21	.28	.37	.36	–		
11. Social lonely	.10	.10	.11	− .14	.21	.18	.19	.23	.36	.61	–	
12. Neg affect	.23	.26	.17	− .10	.29	.19	.32	.53	.47	.49	.36	–
Possible Range	1 − 25	1 − 25	1 − 25	1 − 7	1 − 6	0 − 10	1 − 6	0 − 48	0 − 28	0 − 6	0 − 5	0 − 28
*M*	2.75	5.41	3.56	3.38	1.82	3.72	3.12	25.07	13.26	3.20	2.48	13.21
*SD*	2.70	3.84	3.82	1.46	.99	2.69	1.37	11.40	6.50	2.17	1.79	6.14
Median	1.67	4.50	1.83	3.20	1.50	3.60	3.00	25.00	13.00	3.00	2.00	13.00
Skew	.84	− .19	.68	.51	.64	.34	.15	.09	.05	− .14	.01	.02
*SE* of Skew	.03	.03	.03	.03	.03	.03	.03	.03	.03	.03	.03	.03
Kurtosis	− .15	− .77	− .73	− .51	− .78	− .85	− .92	− .84	− .75	− 1.40	− 1.38	− .59
*SE* of Kurtosis	.06	.06	.06	.06	.06	.06	.06	.06	.06	.06	.06	.06
Omega (ω)	.83	.83	.79	.82	.91	.82	.85	.93	.86	.87	.86	.87

Discrim/harass = everyday discrimination/harassment, Microaggress = microaggressions, Family reject = family rejection, Comm involve = community involvement, IH = internalized homonegativity, Stigma = stigma preoccupation, Social inhib = social inhibition, Social lonely = social loneliness, Emo lonely = emotional loneliness, Neg affect = negative affectivity. Correlations, skewness, and kurtosis for everyday discrimination/harassment microaggressions, family rejection, and internalized homonegativity are based on log-transformed values. For all correlations, *p* < .001

### Measurement Model

All indicators loaded strongly onto their respective factors (> .70, with most over .80). Only one indicator of marginalization (family rejection) had a somewhat lower factor loading (0.63), although this is unsurprising because the other two indicators (everyday discrimination/harassment and microaggressions) pertain to marginalization across all settings, not specifically to family.

Overall, the measurement model had a strong fit to the data: χ^2^(216) = 1846.53, *p* < .001; CFI = .99; TLI = .98; SRMR = .022; RMSEA = .031, 90% CI [.030, .032]. Nonetheless, 19% of the standardized residual covariances were > 2.58. Although fairly well distributed, several of these residuals were concentrated around the second and third emotional loneliness parcels. Modification indices suggested adding a correlation between the error terms of these two parcels. As items with similar wording were found in both parcels (“I miss having people around me” and “I miss the pleasure of the company of others”), we felt that adding the correlation was justified. After this modification, model fit improved: *χ*^2^(215) = 1516.12, *p* < .001; CFI = .99; TLI = .99; SRMR = .020; RMSEA = .028, 90% CI [.026, .029]. Ten percent of residual covariances remained over 2.58; however, when using our smaller samples, almost all were within normal range and the larger ones were broadly scattered, but some still clustered around the third emotional loneliness parcel. Modification indices did not suggest further substantive changes, so we were satisfied with our minimally adjusted measurement model.

### Structural Model

The initial model was an acceptable fit to the data: *χ*^2^(503) = 5093.05, *p* < .001; CFI = .97; TLI =  .94; SRMR = .024; RMSEA = .034, 90% CI [.033, .035]. However, inspection of standardized residual covariances and modification indices suggested correlating the error terms of all three proximal stress factors. Because of the conceptual and empirical similarity between the factors, we felt it was appropriate to do so. Following these adjustments, residual covariances > 2.58 declined to 7% and were less than 3% in our smaller samples. As modification indices suggested no other substantive areas to improve fit, we made no further adjustments.

The revised model fit the data well: *χ*^2^(500) = 3092.15, *p* < .001; CFI = .98; TLI = .97; SRMR = .015; RMSEA = .026, 90% CI [.025, .027]. We decomposed the RMSEA values for the measurement and structural models. RMSEA for the structural model was .024, 90% CI [.023, .025]. Thus, we have confidence that the fit indices for the entire model reflected goodness of fit for *both* the measurement and structural models. In addition, model fit and patterns of association were similar with and without the inclusion of the top 5% and 10% of multivariate outliers.

### Structural Relationships

**Fig. 2:**
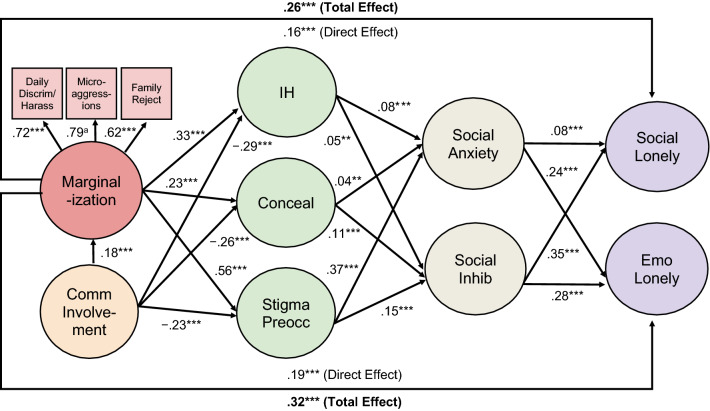
Structural equation model linking marginalization and loneliness*. Notes:* Daily Discrim/Harass = everyday discrimination/harassment, Family Reject = family rejection, Comm Involvement = community involvement, IH = internalized homonegativity, Conceal = concealment, Stigma Preocc = stigma preoccupation, Social Inhib = social inhibition, Social Lonely = social loneliness, Emo Lonely = emotional loneliness. For ease of presentation, direct associations between community involvement/proximal stress and both forms of loneliness are not shown, nor are direct associations between marginalization/community involvement and social anxiety/inhibition (see Table 3 for coefficients). The following correlated residuals (essentially factor correlations) are also not shown: internalized homonegativity and concealment (*r* = *.*37, *p* < .001), internalized homonegativity and stigma preoccupation (*r* = *.*51, *p* < .001), concealment and stigma preoccupation (*r* = *.*39, *p* < .001), social anxiety and social inhibition (*r* = *.*35, *p* < .001), and social and emotional loneliness (*r* = .74, *p* < .001). All values are standardized regression coefficients. ^a^ Strongest path fixed to 1.0 for statistical identification, thus *p*-value cannot be computed. ***p < .001, **p < .01

Hypotheses 1–4 were generally supported, as seen in Fig. 2 and Table 3. Total associations between marginalization and loneliness were positive (Hypothesis 1). Based on empirically derived guidelines for interpreting effect sizes in social psychology (Lovakov & Agadullina, 2021), these associations were moderate: for social loneliness, β = .26, *p* < .001, 95% CI [.22, .29]; for emotional loneliness, β = .32, *p* < .001, 95% CI [.28, .35]. Marginalization was also positively associated with all three proximal stressors: internalized homonegativity, concealment, and, especially, stigma preoccupation (Hypothesis 2). Associations were moderate to large. In turn, stigma preoccupation showed a positive, moderate association with social anxiety, and a smaller positive association with inhibition (Hypothesis 3). The other two proximal stressors—internalized homonegativity and concealment—were also positively associated with social anxiety and inhibition, but to a lesser extent. In mixed support of Hypothesis 3, internalized homonegativity had a small, positive association with both social and emotional loneliness, while concealment had a small, positive association with social loneliness, but a negligible positive association with emotional loneliness. In opposition to Hypothesis 3, there were small, non-significant *negative* associations between stigma preoccupation and both social and emotional loneliness. (For simplicity, direct associations between proximal stress and loneliness are only reported in Table 3, not Fig. 2). Finally, supporting Hypothesis 4, social anxiety and inhibition were positively associated with both social and emotional loneliness. These relationships were moderate, with the exception of the relationship between social anxiety and social loneliness, which was small.

**Table 3:** Direct associations (standardized)

Predictor	Outcome	β	95% CI		*p*
			Lower	Upper	
Marginalization	Social loneliness	.16	.12	.20	< .001
	Emotional loneliness	.19	.15	.23	< .001
	Internalized homonegativity	.33	.30	.36	< .001
	Concealment	.23	.20	.26	< .001
	Stigma preoccupation	.56	.53	.58	< .001
	Social anxiety	.04	.01	.08	.019
	Social inhibition	.01	− .04	.04	.932
Internalized homonegativity	Social anxiety	.08	.05	.11	< .001
	Social inhibition	.05	.02	.09	.002
	Social loneliness	.09	.05	.12	< .001
	Emotional loneliness	.13	.10	.17	< .001
Concealment	Social anxiety	.04	.01	.07	.006
	Social inhibition	.11	.07	.14	< .001
	Social loneliness	.07	.03	.10	< .001
	Emotional loneliness	.01	− .02	.04	.671
Stigma preoccupation	Social anxiety	.37	.33	.41	< .001
	Social inhibition	.15	.11	.19	< .001
	Social loneliness	− .03	− .08	.01	.124
	Emotional loneliness	− .04	− .08	.01	.098
Social anxiety	Social loneliness	.08	.05	.11	< .001
	Emotional loneliness	.24	.21	.27	< .001
Social inhibition	Social loneliness	.35	.32	.38	< .001
	Emotional loneliness	.28	.25	.30	< .001
Community involvement	Marginalization	.18	.16	.21	< .001
	Internalized homonegativity	− .29	− .31	− .26	< .001
	Concealment	− .26	− .28	− .23	< .001
	Stigma preoccupation	− .23	− .26	− .21	< .001
	Social anxiety	.01	− .01	.04	.324
	Social inhibition	− .14	− .17	− .11	< .001
	Social loneliness	− .10	− .14	− .07	< .001
	Emotional loneliness	− .08	− .11	− .05	< .001

As shown in Tables 4 and 5, indirect associations between marginalization and loneliness were mostly in the expected directions, supporting Hypotheses 5 and 6. Although individual associations were small in absolute terms, in combination they amounted to 37% of the total association between marginalization and social loneliness, and 41% of the total association between marginalization and emotional loneliness. Stigma preoccupation played a key role in the indirect associations: marginalization was positively associated with both forms of loneliness via stigma preoccupation and social anxiety, and via stigma preoccupation and social inhibition. In addition to these positive serial associations involving social anxiety/inhibition, there were small, non-significant negative associations between marginalization and both forms of loneliness via stigma preoccupation alone. Marginalization was also positively associated with both forms of loneliness via internalized homonegativity alone, and positively associated with social loneliness via concealment alone. Other indirect associations were consistent with hypotheses, but much smaller.

**Table 4:** Indirect associations between marginalization and social loneliness

Indirect Association	β	*B*	95% CI		*p*	Relative magnitude
			Lower	Upper		
*Serial indirect associations*						
Marginalization $\to$ Stigma Preocc $\to$ Inhibition $\to$ Soc Loneliness	.029	.080	.057	.106	< .001	11%
Marginalization $\to$ Stigma Preocc $\to$ Soc Anxiety $\to$ Soc Loneliness	.017	.047	.028	.066	< .001	7%
Marginalization $\to$ Concealment $\to$ Inhibition $\to$ Soc Loneliness	.008	.023	.016	.032	< .001	3%
Marginalization $\to$Concealment $\to$ Soc Anxiety $\to$ Soc Loneliness	.001	.002	.001	.004	.004	< 1%
Marginalization $\to$ IH $\to$ Inhibition $\to$ Soc Loneliness	.006	.017	.007	.029	.002	2%
Marginalization $\to$ IH $\to$ Soc Anxiety $\to$ Soc Loneliness	.002	.006	.003	.010	< .001	1%
*Simple indirect associations*						
Marginalization $\to$ IH $\to$ Soc Loneliness	.030	.082	.050	.116	< .001	12%
Marginalization $\to$ Stigma Preocc $\to$ Soc Loneliness	− .019	− .053	− .125	.013	.125	− 8%
Marginalization $\to$ Concealment $\to$ Soc Loneliness	.015	.042	.021	.062	< .001	6%
Marginalization $\to$ Soc Anxiety $\to$ Soc Loneliness	.004	.010	.002	.020	.013	1%
Marginalization $\to$ Inhibition $\to$ Soc loneliness	.0003	.001	− .037	.040	.934	< 1%

Stigma Preocc = stigma preoccupation, IH = internalized homonegativity, Soc Anxiety = social anxiety, Inhibition = social inhibition, Soc Loneliness = social loneliness. 95% confidence intervals are for unstandardized betas (*B*). Relative magnitude, a suggested effect size measure, is calculated by dividing each unstandardized indirect association by the total unstandardized association between marginalization and social/emotional loneliness. Associations ordered by size. Percentages rounded

**Table 5:** Indirect associations between marginalization and emotional loneliness

Indirect association	β	*B*	95% CI		*p*	Relative magnitude
			Lower	Upper		
*Serial indirect associations*						
Marginalization $\to$ Stigma Preocc $\to$ Soc Anxiety $\to$ Emo Loneliness	.049	.128	.106	.152	< .001	15%
Marginalization $\to$ Stigma Preocc $\to$ Inhibition $\to$ Emo Loneliness	.023	.061	.043	.081	< .001	7%
Marginalization $\to$ Concealment $\to$ Inhibition $\to$ Emo Loneliness	.007	.018	.012	.025	< .001	2%
Marginalization $\to$ Concealment $\to$ Soc Anxiety $\to$ Emo Loneliness	.002	.006	.002	.010	.005	1%
Marginalization $\to$ IH $\to$ Soc Anxiety $\to$ Emo Loneliness	.006	.016	.010	.023	< .001	2%
Marginalization $\to$ IH $\to$ Inhibition $\to$ Emo Loneliness	.005	.013	.005	.022	.002	2%
*Simple indirect associations*						
Marginalization $\to$ IH $\to$ Emo Loneliness	.045	.117	.087	.149	< .001	14%
Marginalization $\to$ Stigma Preocc $\to$ Emo Loneliness	− .020	− .053	− .117	.009	.100	− 6%
Marginalization $\to$ Soc Anxiety $\to$ Emo Loneliness	.010	.027	.005	.049	.019	3%
Marginalization $\to$ Concealment $\to$ Emo Loneliness	.002	.004	− .015	.022	.663	< 1%
Marginalization $\to$ Inhibition $\to$ Emo Loneliness	.0003	.001	− .028	.031	.931	< 1%

Stigma Preocc = stigma preoccupation, IH = internalized homonegativity, Soc Anxiety = social anxiety, Inhibition = social inhibition, Emo Loneliness = emotional loneliness. 95% confidence intervals are for unstandardized betas (*B*). Relative magnitude, a suggested effect size measure, is calculated by dividing each indirect association by the total association between marginalization and social/emotional loneliness. Associations ordered by size. Percentages rounded

Considering both direct and indirect associations plus covariates, our model accounted for 25% of variance in social loneliness and 35% of variance in emotional loneliness. The model also accounted for 23% of variance in internalized homonegativity, 21% in concealment, 38% in stigma preoccupation, 30% in social anxiety, and 15% in social inhibition.

### Role of Community Involvement

As shown in Fig. 2 and Table 3, there was a small positive association between community involvement and marginalization, as expected (Hypothesis 7). There were also moderate negative associations between community involvement and all three proximal stress factors, and smaller negative associations with both forms of loneliness (Hypothesis 8). Total associations (direct and indirect) between community involvement and loneliness were small: for social loneliness, β =  −.18, *p* < .001, 95% CI [−.21, −.15]; for emotional loneliness, β =  −.13, *p* < .001, 95% CI [−.16, −.10]. About 45% of the total association between community involvement and social loneliness was indirect, as was 40% of the total association between community involvement and emotional loneliness.

After including interaction terms between community involvement and other latent factors, model fit remained strong, albeit slightly weaker than the original model because our chosen fit statistics penalize for model complexity: *χ*^2^(2282) = 17,412.26, *p* < .001; CFI = .96; TLI = .95; RSMR = .026; RMSEA = .029, 90% CI [.029, .030]. Several interactions were statistically significant and in the expected directions, but they were fairly small, providing muted support for Hypothesis 9. For people higher in community involvement, interaction terms indicated somewhat weaker associations between marginalization and internalized homonegativity (interaction β =  −.06, *p* < .001), concealment (interaction β =  −.03, *p* = .039), and stigma preoccupation (interaction β =  −.03, *p* = .007). There were also weaker associations between stigma preoccupation and social anxiety (interaction β =  −.07, *p* = .045), and between social inhibition and social loneliness (interaction β =  −.04*, p* = .020). However, in contrast to Hypothesis 9, greater community involvement was associated with somewhat stronger relationships between concealment and social anxiety (interaction β = .03, *p* = .062). Community involvement did not significantly moderate the direct associations between marginalization and emotional loneliness (β = .01, *p* = .822), or between marginalization and social loneliness (β = .01, *p* = .755). Table 6 lists simple slopes for high, average, and low community involvement.

**Table 6:** Simple slopes as a function of differing levels of community involvement

Relationship	Level of community involvement	β	*B*	95% CI		*p*
				Lower	Upper	
Marginalization $\to$ Internalized homonegativity	Low Average High	.400 .335 .270	.314 .263 .212	.278 .237 .181	.356 .289 .242	< .001 < .001 < .001
Marginalization $\to$ Concealment	Low Average High	.261 .231 .201	2.586 2.288 1.990	2.123 1.989 1.633	3.064 2.605 2.345	< .001 < .001 < .001
Marginalization $\to$ Stigma preoccupation	Low Average High	.591 .557 .523	3.088 2.909 2.731	2.872 2.742 2.514	3.310 3.070 2.928	< .001 < .001 < .001
Concealment $\to$ Social anxiety	Low Average High	.010 .044 .075	.004 .017 .029	− .013 .006 .011	.021 .027 .049	.687 .002 .002
Stigma Preoccupation $\to$ Social anxiety	Low Average High	.449 .366 .284	.327 .267 .207	.264 .238 .127	.405 .295 .273	< .001 < .001 < .001
Social Inhibition $\to$ Social loneliness	Low Average High	.392 .347 .301	.309 .273 .237	.272 .251 .198	.347 .295 .276	< .001 < .001 < .001

High = one standard deviation above the mean in community involvement. Average = mean level of community involvement. Low = one standard deviation below the mean in community involvement. 95% confidence intervals are for unstandardized betas (*B*)

### Role of Dispositional Negative Affectivity

**Fig. 3:**
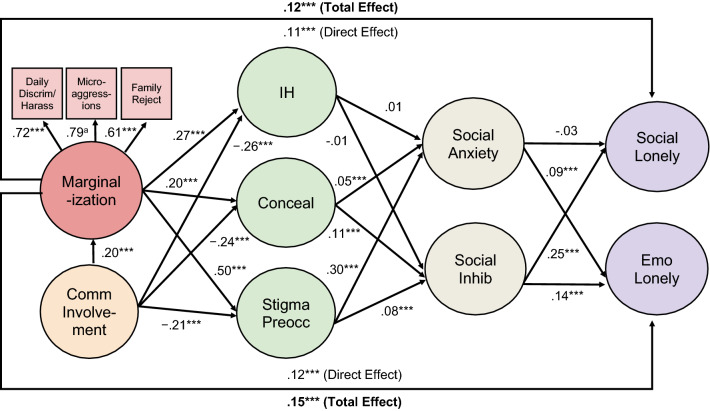
Structural equation model linking marginalization and loneliness, adjusted for dispositional negative affectivity*. Notes:* Daily Discrim/Harass = everyday discrimination/harassment, Family Reject = family rejection, Comm Involvement = community involvement, IH = internalized homonegativity, Conceal = concealment, Stigma Preocc = stigma preoccupation, Social Inhib = social inhibition, Social Lonely = social loneliness, Emo Lonely = emotional loneliness. For ease of presentation, direct associations between community involvement/proximal stress and both forms of loneliness are not shown, nor are direct associations between marginalization/community involvement and social anxiety/inhibition. The following correlated residuals (essentially factor correlations) are also not shown: internalized homonegativity and concealment (*r* = *.*36, *p* < .001), internalized homonegativity and stigma preoccupation (*r* = *.*49, *p* < .001), concealment and stigma preoccupation (*r* = *.*38, *p* < .001), social anxiety and social inhibition (*r* = *.*17, *p* < .001), and social and emotional loneliness (*r* = .72, *p* < .001). All values are standardized regression coefficients. ^a^ Strongest path fixed to 1.0 for statistical identification, thus *p*-value cannot be computed. ***p < .001, **p < .01

After adjusting for general negative affectivity (a latent factor with three indicators, all loading over .80), the model was still a good fit to the data: *χ*^2^(601) = 4271.84; CFI = .98; TLI = .96; SRMR = .016; RMSEA = .028, 90% CI [.027, .029]. However, supporting Hypothesis 10, total associations between marginalization and loneliness declined by about half (54% reduction for social loneliness and 53% reduction for emotional loneliness) (Fig. 3). Direct associations with social and emotional loneliness declined by 31% and 37%, while indirect associations declined by 90 and 77%. When using frequency-only measures of marginalization, the inclusion of negative affectivity reduced the total associations between marginalization and both forms of loneliness to a slightly lesser extent (47% reduction for social loneliness and 50% reduction for emotional loneliness). The adjusted model accounted for 30% of variance in social loneliness, 43% in emotional loneliness, 26% in internalized homonegativity, 22% in concealment, 40% in stigma preoccupation, 46% in social anxiety, and 33% in social inhibition.

## Discussion

### Relationships Between Minority Stress, Social Anxiety/Inhibition, and Loneliness

Our findings provide additional support for minority stress theory by demonstrating direct and indirect associations between marginalization, proximal stress, and loneliness. Our findings also extend Hatzenbuehler’s (2009) psychological mediation framework by examining the roles of social anxiety and inhibition in the relationship between minority stress and loneliness.

Notably, we found that marginalization was more strongly associated with stigma preoccupation than with concealment and internalized homonegativity. While marginalization likely increases fear of judgment and rejection, it might not necessarily increase self-hatred. Indeed, for some sexual minorities, it may even generate feelings of pride, countering negative impacts. In contrast, some people may internalize negative attitudes without having been marginalized themselves (e.g., by observing others being marginalized). Similarly with concealment, some who have been marginalized might react by hiding their sexual orientation, whereas others might feel even more motivated to be “out and proud.” These contrasting reactions may explain why the correlations between marginalization, internalized homonegativity, and concealment were relatively smaller.

Turning to associations between proximal stress and social anxiety/inhibition, we found that stigma preoccupation was most strongly associated with these two variables. It also played played the most prominent role in the indirect associations between marginalization and loneliness. The role of concealment may have been muted because it is not necessarily negative for everyone. While some who conceal their orientation might become very self-conscious and constantly fear discovery (Pachankis, 2007), others may find that it has less impact on social anxiety because they are able to successfully avoid detection and, thus, marginalization (e.g., gender-conforming gay men). For its part, internalized homonegativity relates more to feelings and attitudes about the self rather than specific fear of evaluation by others.

We also found small direct associations between internalized homonegativity and both forms of loneliness, as well as between concealment and social loneliness. This is not surprising; internalized beliefs that non-heterosexual relationships are inferior or dysfunctional may reduce trust, commitment, and intimacy; and may increase relationship conflict and dissatisfaction (Cao et al., 2017; Downs, 2012; Doyle & Molix, 2015; Frost & Meyer, 2009). These beliefs might also foster unrealistically high relationship standards (Downs, 2012). For its part, concealment can contribute to social loneliness by increasing real and perceived distance from others and curtailing one’s social network.

In contrast to most of the positive associations we found between minority stress and loneliness, there were some *negative* direct associations between stigma preoccupation and both forms of loneliness, which produced corresponding negative indirect associations between marginalization and loneliness. Although these associations were quite small and not statistically significant, they raise the possibility of countervailing relationships between marginalization and loneliness which should be explored in future studies. On one hand, marginalization could lead to loneliness if it increases social anxiety and inhibition to the point of interfering with relationships, as suggested by our model. On the other hand, marginalization might reduce loneliness to an extent by motivating some stigma-preoccupied individuals who are not anxious/inhibited to associate with people who are especially accepting and supportive.

Turning to relations between social anxiety, inhibition, and loneliness, is it perhaps unsurprising that inhibition was more strongly associated with social versus emotional loneliness. The former pertains to a broader social network, whereas the latter pertains to feelings of intimacy and attachment. Inhibited people may have trouble building a broad social network, especially in urban gay areas that cater to extroverts. It is also notable that social anxiety had a much stronger relationship with emotional versus social loneliness. Social anxiety relates to fears about social rejection; while these fears can apply in all situations, they may be more salient and consequential in the context of close rather than distant relationships (e.g., dating). This may be especially so for sexual minorities, for whom a history of rejection may have kindled fears about approval, trust, intimacy, and fidelity (Cao et al., 2017; Downs, 2012; Doyle & Molix, 2016; Frost & Meyer, 2009; Hobbes, 2017). Another reason for the strong relation between social anxiety and emotional loneliness is that they both share variance with negative affectivity (discussed below).

Overall, the total associations between marginalization and loneliness were moderate, based on empirically derived guidelines for interpreting effect sizes in social psychology (Lovakov & Agadullina, 2021). This is unsurprising given the multitude of reasons people may feel lonely (de Jong Gierveld et al., 2018; Elmer, 2018; Lim et al., 2020). Countervailing relationships between minority stress and loneliness, as suggested above, may also have limited the effect sizes. In addition, our sample reflected diverse experiences in 85 countries. While we controlled for broad geographic region, we did not control for specific country of residence. Associations in our model may be modest in some countries but stronger in others, which we will explore in another study using these same data. Finally, even moderate associations between minority stress and loneliness can have a serious impact, as loneliness is a well-known risk factor for morbidity and early mortality, especially when chronic (Cacioppo & Cacioppo, 2014; Holt-Lunstad et al., 2015). Of particular concern, some respondents reported very high levels of minority stress, so they may be quite lonely and thus at even greater risk for health problems.

Indirect associations were also small in absolute value, although this is expected when using multiple serial mediators (Walters, 2019). Nonetheless, these associations suggest some of the theoretically and clinically relevant mechanisms by which minority stress may be related to loneliness. It is also important to note that while specific indirect associations may be small on their own, their combined effects can be meaningful, especially if they accumulate and compound over time, or if they impact large numbers of people (Götz et al., 2021).

In total, about 40% of the total relationship between marginalization loneliness was indirect. This could mean there are other unmeasured factors that play a role in the link between marginalization and loneliness (e.g., rumination, emotional dysregulation; Preece et al., 2021). Alternatively, it could mean that most of the relationship between marginalization and loneliness is, in fact, direct (e.g., marginalization directly leads to feeling different, misunderstood, or estranged, even in the absence of internalized homonegativity, concealment, stigma preoccupation, or social anxiety/inhibition).

Loneliness itself could also contribute to marginalization; for example, those who are friendless or who have traits associated with loneliness (e.g., shyness, low self-esteem, passivity) may be targeted for harassment and bullying (Acquah et al., 2016; Pavri, 2015). Loneliness can also increase social withdrawal, self-focus, irritability, hostility, and other aversive behaviors and emotions that may elicit rejection by others (Cacioppo & Hawkley, 2009; Cacioppo et al., 2017; Mund & Neyer, 2016, 2019; Qualter et al., 2015; Segel-Karpas & Ayalon, 2020; Spithoven et al., 2017; van Winkel et al., 2017). In this sense, marginalization and loneliness are likely mutually reinforcing; future studies using longitudinal data should examine this possibility.

### Is LGBTQ Community Involvement Protective?

Our study also examined the specific role of LGBTQ community involvement. Three findings are notable. First, there was a modest but positive association between community involvement and marginalization, in line with previous studies (Bissonette & Syzmanski, 2019; Chan & Mak, 2021; Foster-Gimbel et al., 2018; Kuyper et al., 2016; Ramirez-Valles et al., 2005; Velez & Moradi, 2016). One reason may be that those who are involved in the community are more open about their sexual orientation, which may increase their risk of marginalization (Bissonette & Syzmanski, 2019; LeBeau & Jellison, 2009). This is consistent with studies finding a positive association between outness and marginalization (e.g., Brewster et al., 2013; Cook et al., 2013; Timmins et al., 2020). Community involvement might also remind a person of their stigmatized status or contribute to a stronger sexual minority identity (LeBeau & Jellison, 2009; Vaughan & Waehler, 2010); this might increase perceptions and identification of marginalization (Ramirez-Valles et al., 2005). Of course, the relationship between community involvement and marginalization may work in the other direction, too: those who experience or perceive marginalization in the first place may be more likely to join the LGBTQ community (e.g., to find support or advocate for LGBTQ rights; Chan & Mak, 2021).

Although community involvement may confer risk for marginalization, our results are also consistent with the suggestion that it can protect against the negative consequences of marginalization, both by reducing the amount of proximal stress, inhibition, and loneliness, as well as buffering the links between these factors and marginalization. (Meyer, 2003). Notably, involvement was negatively associated with both forms of loneliness, with nearly half of this relationship being indirect via negative associations with proximal stress and social inhibition. While this suggests that community involvement plays a protective role, it is also possible that those who are more out and comfortable with their sexuality in the first place—as well as less inhibited—are more likely to get involved in the LGBTQ community.

We also found that the associations between marginalization and proximal stress were somewhat weaker among those who were more community-involved, as were associations between stigma preoccupation and social anxiety, and between social inhibition and social loneliness. Sexual minority peers can provide support to reduce the emotional impact of marginalization; a safe environment that reduces the need for self-monitoring and impression management; encouragement to resist or adaptively reappraise stigma and internalized homonegativity; and positive examples of successful relationships to counter self-defeating beliefs that same-sex relationships are inherently dysfunctional (Cox et al., 2010; Meyer, 2003; Velez & Moradi, 2016; Westefeld et al., 2001). Community involvement may also increase personal control and self-efficacy (Chan & Mak, 2021; Heath & Mulligan, 2008; van Lisdonk & Kuyper, 2015; Wernick et al., 2014); this may reduce passivity and promote an active approach to relationships (e.g., managing social anxiety, meeting new people, resolving conflicts; Newall et al., 2014).

All of these advantages could help to reduce loneliness. So, too, could the more basic functions of LGBTQ community involvement—providing opportunities for socializing and meeting new friends and partners—which likely contributed to the direct associations we observed between community involvement and loneliness. In contrast to our hypotheses, however, community involvement did not appear to moderate the direct associations between marginalization and loneliness. These direct associations may be due partly to a sense of feeling different, misunderstood, or estranged from people outside the LGBTQ community; if so, perhaps community involvement is limited in its ability to reduce these feelings.

While valuable in many respects, community involvement may not benefit everyone to the same extent. First, some people are involved in the community but do not feel close to anyone, hence community involvement does not really protect them from loneliness. Indeed, what matters more in terms of loneliness is the perceived quality of relationships and feeling connected, not merely the presence of others (Hawkley et al., 2008). Second, very high levels of community involvement may take a toll for some people in terms of time, energy, and vicarious exposure to marginalization (Bissonette & Syzmanski, 2019; Kulick et al., 2017). Third, the benefits of community involvement may be counterbalanced by various intra-minority stressors within the community itself. Examples include political conflicts, conformity, racism, bi-negativity, status-seeking, cliquishness, competitiveness, and a focus on youth, appearance, and sex, especially among younger gay men in urban centers and on social media/dating/sex apps (Aggarwal & Gerrets, 2014; Hammack et al., 2021; Heath & Mulligan, 2008; Hobbes, 2017; LeBeau & Jellison, 2009; Lehavot et al., 2009; Mereish et al., 2017; O’Byrne et al., 2014; Pachankis et al., 2020; Parmenter et al., 2020). The countervailing impact of these intra-minority stressors, which we intend to explore in another paper using these same data, may explain why the associations we observed between community involvement and loneliness were fairly small, as were interactions. The last finding of note was that for those higher in community involvement, there were slightly stronger associations between concealment and social anxiety. For people active in the LGBTQ community but not necessarily out to others, their fear of detection may be higher, thus increasing social anxiety in other areas of their life.

### Is Dispositional Negative Affectivity a Confound?

In light of criticism that associations between self-reported marginalization and mental health may be confounded by trait negative affectivity (NA; Bailey, 2020; Lilienfeld, 2017), we controlled for it in our study. After doing so, we found that our model still fit the data well, but total associations between marginalization and loneliness dropped by half. This ruled out the possibility that associations were mostly spurious, but confounding was still conceivable. At the same time, this procedure may have amounted to “over-control”: to the extent that trait NA is caused by actual cumulative experiences of marginalization, controlling for it likely removed some of the variance intrinsic to marginalization and thus underestimated the true relationship between marginalization and loneliness (Lilienfeld, 2017). Similarly, we may have removed the variance in NA caused by proximal stress, anxiety/inhibition, and loneliness, as well as shared variance due to conceptual overlap between these variables. This likely contributed to the large declines in indirect associations. Importantly, we found similar reductions in associations when using frequency-only measures of marginalization that excluded subjective distress evaluations. If confounding were a serious issue, we should have seen more substantial reductions when using these measures, but we did not. Granted, even frequency-only measures can be influenced by NA (e.g., misinterpreting ambiguous events as discrimination), but unlikely to the same extent. We also found no substantial reduction in associations between marginalization and proximal stress, which we would expect if NA were a major confound.

### Practical Implications

Overall, our findings underscore the continuing need to reduce marginalization of sexual minorities. This would likely reduce loneliness, as well as the negative health impact associated with both minority stress (Flentje et al., 2020; Meyer, 2003) and loneliness (Cacioppo & Cacioppo, 2014; Holt-Lunstad et al., 2015). Our findings also suggest possible interventions at the individual level. Clinicians might show clients how minority stress contributes to social anxiety, inhibition, and loneliness. This could help reframe their struggles as a natural response to marginalization rather than an inherent character defect (Velez & Moradi, 2016). Clinicians could also show clients how negative affectivity may compound their perception of, and reaction to, minority stress. Skills could be taught to cope with minority stress, social anxiety, and inhibition, like identifying positive aspects of one’s sexual orientation; checking automatic, yet possibly incorrect assumptions about innocuous or ambiguous social situations; improving emotion regulation in response to marginalization; minimizing avoidance; enhancing assertiveness and communication of needs and emotions; tempering unrealistic relationship standards; and reducing self-focus (Chaudoir et al., 2017; Downs, 2012; Feinstein, 2020; Hart et al., 2020; LeBeau, 2020; Smith et al., 2017).

These interventions are especially relevant to loneliness, which can exacerbate negative affectivity, hypervigilance, social anxiety, withdrawal, passivity, self-focus, negative social appraisals, and hostility (Cacioppo & Hawkley, 2009; Cacioppo et al., 2013, 2017; Lim et al., 2016; Mund & Johnson, 2021; Mund & Neyer, 2019; Qualter et al., 2015; Segel-Karpas & Ayalon, 2020; Spithoven et al., 2017; van Winkel et al., 2017). Indeed, loneliness interventions showing the most promise are those which address maladaptive social cognition (Masi et al., 2011). Of course, care should be taken not to inadvertently pathologize the reactions to minority stress and loneliness. While they can be counterproductive if disproportionate or inflexible (e.g., expecting rejection even in neutral or welcoming situations), they ultimately begin as self-protective mechanisms that are adaptive in some situations (e.g., accurately anticipating or guarding against rejection or victimization in environments known to be hostile to sexual minorities).

Based on our findings, it would seem fruitful to encourage clients to develop stronger relationships with the LGBTQ community, so long as these relationships are healthy and supportive. This may even benefit those who are not particularly outgoing; indeed, the association between inhibition and social loneliness was *lower* for those who were more involved in the community. This is likely because inhibited people can still find a satisfying social network in the LGBTQ community by engaging in activities that do not involve large groups (e.g., being a member of a small support group). Of course, to the extent that minority stress, social anxiety, inhibition, and loneliness may be impeding relationships, these factors should be addressed beforehand; notably, some research finds that community involvement may have diminishing returns for people high in internalized homonegativity (Salfas et al., 2019). For service providers working with groups, they should also remember that loneliness can spread between people by distorting social cognition and reducing prosocial behavior (Cacioppo et al., 2009; Lieberz et al., 2021; Simon & Walker, 2018). These dynamics should be addressed proactively so that loneliness does not threaten the cohesion of groups and their ability to provide support.

For those experiencing minority stress *within* the community (e.g., racism, ageism), it may be useful to help them find additional or alternate sources of support and companionship. Indeed, some sexual minorities find that a mixed social network is healthier for them than relying exclusively on the LGBTQ community (Holt, 2011). This may be especially true for people who feel that their sexuality is not a central part of their identity.

Finally, clinicians should not underestimate the importance of basic health practices that are implicated in minority stress, social cognition, and loneliness. Sleep is a prime example. Longitudinal studies suggest not only that loneliness contributes to poor sleep—perhaps by increasing hypervigilance for social threat—but poor sleep also contributes to loneliness, perhaps by distorting social cognition, increasing emotional reactivity, and motivating social withdrawal (Hom et al., 2020). Given the impact of minority stress, it is not surprising that sexual minorities have more sleep problems compared to others (Patterson & Potter, 2019); helping them improve sleep could buffer the links between minority stress and loneliness.

### Limitations, Strengths, and Future Directions

Our findings should be interpreted in light of several limitations. First, the prevalence of minority stress—especially everyday discrimination/harassment, family rejection, and internalized homonegativity—was quite low. It is possible that those with higher levels of minority stress chose not to participate in our study. Moreover, social media ads were targeted to individuals who had divulged their sexual orientation (or interests related to sexual orientation), which may have biased the sample toward those who are more out/comfortable with their sexual orientation. Indeed, only about 30% of respondents indicated that they actively conceal their sexual orientation from others half of the time or more. Although low levels of marginalization and internalized homonegativity are not uncommon in many studies (e.g., Everett et al., 2009; Mereish & Poteat, 2015; Mohr & Kendra, 2011; van Lisdonk & Kuyper, 2015; Velez & Moradi, 2016), restricted range of responses may have underestimated some associations. Other variables did not exhibit restricted range.

Our data were based on self-report. In addition to the possible confounding role of general negative affectivity, responses were subject to social desirability and retrospective recall bias. Respondents may have under-reported minority stress and loneliness to counter perceptions that sexual minorities are inherently troubled. Conversely, some may have over-reported minority stress to underscore disparities between LGBTQ people and others. Although we focused on marginalization in the past twelve months, even this timeframe is subject to recall bias. Therefore, it would be useful to test our hypotheses using ecological momentary assessments and daily diary studies. It would also be useful to compare associations of loneliness with past-year versus lifetime marginalization (Ejlskov et al., 2020; Lyons et al., 2021).

All scales were administered in English. To minimize comprehension problems, we showed ads to social media users who had indicated that they understood English. Although internal consistency for scale responses was similar across countries and races/ethnicities—suggesting that items were understood in a similar manner—it is inevitable that non-native English speakers had greater difficulty understanding some questions. Moreover, in English-dominant countries, those without English proficiency may be of lower socioeconomic status (e.g., recent immigrants) and thus underrepresented in our survey. Conversely, bilingual respondents in countries where English is not dominant may be of higher SES (e.g., more educated), and thus less representative of the general LGBTQ population. SES is especially relevant to minority stress: not only are lower-SES sexual minorities more likely to be marginalized, they may also be more isolated from the mainstream LGBTQ community and thus more vulnerable to the negative impact of marginalization (McGarrity, 2014). That said, the median score on our income comfort scale was 4 (on a scale of 1–7) and responses were fairly normally distributed, with each level of income comfort well-represented. This suggests there were enough low-SES respondents in our sample. Nevertheless, our model should be compared across SES groups.

Our study had sizeable drop-out (45%), apparently due to survey length. Although this rate is similar to that of other large online studies of sexuality and minority stress (e.g., Community-Based Research Centre, 2017; Meyer et al., 2020; Reimers, 2007), it may have biased our sample. Those who persevered may be unique in terms of minority stress exposure/perception, personality, motivation for participating (e.g., promotion of LGBTQ rights), cognitive ability, and health. Completion was higher among those who were older, White, queer, and pan/polysexual. In addition to being more conscientious, it is possible that older participants were more motivated to finish due to their greater involvement and interest in the LGBTQ community. They were also less likely to complete on a mobile device, resulting in fewer technical problems (e.g., trouble displaying items or pausing and resuming the survey). Similar reasons likely applied to queer and pan/polysexual individuals, who were more involved in the community and, with the exception of queer respondents, were also younger. White respondents likely experienced fewer language problems and were also younger and more likely to complete on a mobile device. It should be noted that the drop-out rate may have been inflated to some extent by users who began on one device and then started over on another (e.g., people who had technical problems on a mobile and switched to desktop). Their responses would appear to come from two different people, with the first set appearing to come from a non-completer. Unfortunately, it was not possible to determine how many of these respondents there were.

Finally, although SEM represents relationships as causal, our data were cross-sectional, and SEM by itself cannot prove causality. Our hypothetical model was consistent with our data, but alternate models proposing different causal mechanisms could also be a good fit to our data (e.g., models in which loneliness is hypothesized to increase social anxiety or perceptions of marginalization) (Kline, 2016). Although past research supports prospective links between some of our variables (e.g., Hatzenbuehler et al., 2008; Jackson et al., 2019; Pachankis & Bernstein, 2012), additional longitudinal studies are needed to disentangle causal mechanisms. Indeed, temporal relationships proposed by our model are likely more complicated than in the usual formulations of minority stress theory, and may be bidirectional. For example, there is evidence that proximal stress can precede perceived marginalization (e.g., Douglass & Conlin, 2020). Experimental studies could also be instructive (e.g., manipulating loneliness to see how it affects perceptions of minority stress, especially in ambiguous social situations).

Social media yielded a large, diverse sample from 85 countries. Minority stress studies have typically focused on single countries, usually in North America, Europe, and Australia. By contrast, many of our respondents were from countries that have received less attention, like South Africa and New Zealand, and one-third were from non-Western regions. This allowed us to capture a wide range of experiences with minority stress, given the substantial cross-national variation in attitudes toward sexual minorities (e.g., European Union Agency for Fundamental Rights, 2020). Notably, even after controlling for broad geographic region, our models were still robust, with many medium-size associations between minority stress, social anxiety/inhibition, and loneliness. This suggests that minority stress uniquely contributes to mental health problems and loneliness, independent of one’s location. These results are consistent with an international study of gay and bisexual men, which found that minority stress theory is a sound cross-cultural model for understanding life satisfaction (Sattler & Lemke, 2019). To extend our findings, it would be fruitful to compare our model by specific countries. It would also be useful to compare it by ethnoracial background, not simply with respect to respondents’ self-identity, but also their perception of whether they are an ethnoracial minority in their country and whether others perceive them that way. Our single ethnoracial question did not permit this nuanced analysis. Comparisons by gender, sexual orientation, and age would also be informative.

Our study extends minority stress theory and the psychological mediation framework beyond commonly studied mental health and behavioral outcomes (e.g., depression, general anxiety, substance use) and into more interpersonal domains like loneliness. We also examined basic psychological processes that might underlie the relationship between minority stress and loneliness, and we considered both overt and subtle marginalization. We also examined the possible confounding role of dispositional negative affectivity, which many self-report studies overlook.

In contrast to research focusing mostly on the negative impact of minority stress, we also addressed a protective factor: involvement in the LGBTQ community. Thanks to social media, we reached people with widely varying levels of community involvement. This differs from many other studies, which have recruited people from LGBTQ venues and support organizations—people who are often highly involved in the community. This can be a source of bias because these individuals may have unique characteristics and higher rates of actual or perceived marginalization (Bissonette & Syzmanski, 2019; Kuyper et al., 2016; Meyer & Wilson, 2009; Pachankis et al., 2020).

Moving forward, studies should examine other types of marginalization like vicarious discrimination, additional proximal stress factors like difficulty developing a positive sexual identity, and other basic psychological factors like rumination and emotional dysregulation (e.g., Dyar et al., 2018; Sarno et al., 2020; Timmins et al., 2020). Finally, personality traits should be given more attention. Although we examined general negative affectivity, we did so only as a control variable. While this helped rule out the possibility that results were largely spurious, it did not permit an examination of negative affectivity as a precursor, mediator, or moderator in our model (Spector et al., 2000). Doing so would further extend minority stress theory and elucidate the specific mechanisms by which minority stress may lead to the development and reinforcement of loneliness.

## Data Availability

Available by request from the first author.
